# Beyond the bundle - journey of a tertiary care medical intensive care unit to zero central line-associated bloodstream infections

**DOI:** 10.1186/cc12551

**Published:** 2013-03-04

**Authors:** Matthew C Exline, Naeem A Ali, Nancy Zikri, Julie E Mangino, Kelly Torrence, Brenda Vermillion, Jamie St Clair, Mark E Lustberg, Preeti Pancholi, Madhuri M Sopirala

**Affiliations:** 1 Division of Pulmonary, Allergy, Critical Care, and Sleep Medicine, Department of Internal Medicine, Ohio State University Wexner Medical Center, 201 Davis Heart & Lung Research Institute, 473 West 12th Ave, Columbus, OH, 43210, USA; 2 Department of Clinical Epidemiology, Ohio State University Wexner Medical Center, 410 West 10th Ave, Columbus, OH, 43210, USA; 3 Division of Infectious Diseases, Department of Internal Medicine, Department of Clinical Epidemiology, Ohio State University Wexner Medical Center, 410 West 10th Ave, Columbus, OH, 43210, USA; 4 Department of Nursing, Ohio State University Wexner Medical Center, 410 West 10th Ave, Columbus, OH, 43210, USA; 5 Division of Infectious Diseases, Department of Internal Medicine, Ohio State University Wexner Medical Center, 410 West 10th Ave, Columbus, OH, 43210, USA; 6 Department of Pathology, Ohio State University Wexner Medical Center, 1492 East Broad St Columbus, OH, 43205, USA

## Abstract

**Introduction:**

We set a goal to reduce the incidence rate of catheter-related bloodstream infections to rate of <1 per 1,000 central line days in a two-year period.

**Methods:**

This is an observational cohort study with historical controls in a 25-bed intensive care unit at a tertiary academic hospital. All patients admitted to the unit from January 2008 to December 2011 (31,931 patient days) were included. A multidisciplinary team consisting of hospital epidemiologist/infectious diseases physician, infection preventionist, unit physician and nursing leadership was convened. Interventions included: central line insertion checklist, demonstration of competencies for line maintenance and access, daily line necessity checklist, and quality rounds by nursing leadership, heightened staff accountability, follow-up surveillance by epidemiology with timely unit feedback and case reviews, and identification of noncompliance with evidence-based guidelines. Molecular epidemiologic investigation of a cluster of vancomycin-resistant *Enterococcus faecium *(VRE) was undertaken resulting in staff education for proper acquisition of blood cultures, environmental decontamination and daily chlorhexidine gluconate (CHG) bathing for patients.

**Results:**

Center for Disease Control/National Health Safety Network (CDC/NHSN) definition was used to measure central line-associated bloodstream infection (CLA-BSI) rates during the following time periods: baseline (January 2008 to December 2009), intervention year (IY) 1 (January to December 2010), and IY 2 (January to December 2011). Infection rates were as follows: baseline: 2.65 infections per 1,000 catheter days; IY1: 1.97 per 1,000 catheter days; the incidence rate ratio (IRR) was 0.74 (95% CI = 0.37 to 1.65, *P *= 0.398); residual seven CLA-BSIs during IY1 were VRE *faecium *blood cultures positive from central line alone in the setting of findings explicable by noninfectious conditions. Following staff education, environmental decontamination and CHG bathing (IY2): 0.53 per 1,000 catheter days; the IRR was 0.20 (95% CI = 0.06 to 0.65, *P *= 0.008) with 80% reduction compared to the baseline. Over the two-year intervention period, the overall rate decreased by 53% to 1.24 per 1,000 catheter-days (IRR of 0.47 (95% CI = 0.25 to 0.88, *P *= 0.019) with zero CLA-BSI for a total of 15 months.

**Conclusions:**

Residual CLA-BSIs, despite strict adherence to central line bundle, may be related to blood culture contamination categorized as CLA-BSIs per CDC/NHSN definition. Efforts to reduce residual CLA-BSIs require a strategic multidisciplinary team approach focused on epidemiologic investigations of practitioner- or unit-specific etiologies.

## Introduction

Healthcare-associated infections (HAI) are a significant cause of morbidity and mortality for hospitalized patients accounting for approximately 100,000 deaths yearly in the United States [1]. Though intensive care unit (ICU) beds make up the minority of hospital beds nationwide, they account for the highest burden of nosocomial infections [2]. Specifically, in the ICU, central line-associated bloodstream infections (CLA-BSI) account for much of the excess morbidity, health cost expenditures, and mortality associated with nosocomial infections [3-6].

The risk of developing a CLA-BSI depends on a variety of factors such as the duration of catheterization, location of catheter, and type of ICU to which a patient is admitted [4,5,7]. Evidence-based interventions effective in combating CLA-BSIs include: using chlorhexidine skin preparation and maximal sterile barriers (MSB) during insertion of central venous catheters (CVC), use of checklists for insertion, using the subclavian or internal jugular vein instead of the femoral vein, and daily review of line necessity [5-13].

These techniques have been validated in the literature and put together in a 'bundle', which was installed by the Institute for Healthcare Improvement (IHI) to help providers deliver more consistent care [14-18]. However, many of these studies have focused primarily on the insertion of the central line [15,17-19] rather than ongoing line maintenance. Other studies using compliance coupled with adherence to safe line maintenance standards and prompt removal, despite improvement in CLA-BSI rates, have not necessarily documented rates below the National Health Safety Network (NHSN) benchmarks for CLA-BSI and certainly continue to show rates of CLA-BSI above the ultimate goal of 'near-zero' [15,16]. There have been suggestions that the high sensitivity of the CLA-BSI surveillance definition by the Center for Disease Control (CDC)/National Health Safety Network (NHSN) leads to categorization of positive blood cultures as CLA-BSI when they may not actually be related to infections, rather contamination [20]. Some authors have made suggestions that zero CLA-BSI may not be realistic at all [21]. Regardless of the cause of these positive blood cultures, they have a potential to lead to increased antibiotic use, removal of catheters with placement of new catheters and even increased hospital length of stay.

We implemented a systematic team approach with very aggressive interventions surrounding the IHI CLA-BSI bundle resulting in marginal success toward the target of reducing infections to near-zero in our ICU. However, our innovative approach toward implementing these interventions allowed us to reexamine the central line bundle efficacy and augment our process improvement strategy with accessory interventions as our unit's journey progressed to a near-zero rate of CLA-BSI.

## Materials and methods

### Design overview

This was an observational cohort study that used historical controls. The project was deemed as quality improvement by the Institutional Review Board of this organization and need for research approval and informed consent was waived.

### Setting and participants

The study setting was a 25-bed medical ICU located in 1200-bed tertiary care academic hospital at the Ohio State University Wexner Medical Center. All patients admitted or transferred into the unit were included in the intervention. The patient population did not include surgical ICU patients. The nursing to patient ratio averaged 1:1.5 and varied between 1:1 and 1:2 as patient acuity mandated.

### Routine surveillance for CLA-BSI

We measured CLA-BSI rates during the following time periods: baseline (January 2008 to December 2009), intervention year (IY) 1 (January to December 2010), and intervention year 2 (January to December 2011). All blood cultures obtained from patients admitted to the ICU were reviewed by the infection preventionist and all suspected CLA-BSI were confirmed by an epidemiologist/infectious diseases physician utilizing the definition put forth by the CDC through the NHSN [22]. We defined a central line as a catheter that ends in the superior or inferior vena cava at or near the heart. Specific lines present in our population included peripherally inserted central catheters (PICC), central venous catheters (CVC), and pulmonary artery catheters (PA). Arterial lines were not included in CLA-BSI surveillance per NHSN definition. However, we have not noticed any central arterial line infections during our routine surveillance. Total patient days were calculated daily by number of patients on the ICU service census at midnight. The presence of at least one central line in a patient was counted as one catheter day in accordance with the NHSN guidelines [22]. There were no changes to the epidemiology staff during the intervention period, nor were there any changes to the CLA-BSI definition utilized over the course of the study.

### Interdisciplinary team formation - December 2009

A multidisciplinary team that included an infectious diseases physician and infection preventionist, ICU medical directors (critical care physicians), nurse manager and clinical nurse specialists (CNS) was convened. Each individual's role in the performance improvement process was clearly defined in the initial meetings.

### Interventions related to central line bundle - January 2010

Several interventions focused on the central line insertion bundle, dressing maintenance and line access practices were simultaneously introduced or reemphasized to ICU physicians and nurses in January 2010. This marked the beginning of the intervention period.

1. At the start of each rotation, education was reinforced to all house staff to use the Vascular Access Selection Criteria to ensure proper selection of catheter site with emphasis on internal jugular or subclavian placement [23]. This education was part of a refresher course in the hospital simulation laboratory on line placement and sterile technique.

2. Lines in the ICU were placed by resident physicians, critical care or nephrology fellows, critical care attending physicians, or the hospital PICC insertion nursing team. Ultrasound was used to place nonemergent central lines.

3. The continued need for a CVC was reviewed daily, during interdisciplinary ICU rounds by the critical care fellow as part of the daily goals checklist, with removal of the catheter wherever possible [3-5]. In addition, the critical care fellow and the CNS reviewed line necessity during quality rounds that were conducted each afternoon.

4. Nurses placed peripheral intravenous catheters with ultrasound guidance wherever possible to avoid CVC placement and to facilitate removal.

5. Removal within 24 hours of all CVCs placed emergently, that is 'code lines' or any line placed without maximum sterile barrier precautions (sterile gown, sterile gloves, full-size sterile drape, face mask, cap, and chlorhexidine skin preparation solution). A label was used to identify these catheters as emergently placed central lines.

6. CNS led mandatory demonstration session for dressing change and proper line access on a manikin for all nursing staff at the beginning of the study. During this session, all nursing staff was assessed for competence in their dressing change and line access techniques. Chlorhexidine gluconate (CHG) Tegaderm™ dressings were used on all central lines from the beginning of IY1. Nursing performance was evaluated annually by unit management staff as part of annual mandatory education.

7. A CVC insertion checklist, with all requirements to comply with the sterile procedure for CVC placement, was attached to all central line kits. All CVCs placed were antimicrobial catheters. Nursing staff was instructed to use the checklist at the time of line insertion. All providers in the room were required to wear sterile cap, mask, and gloves. Nurses were empowered to stop procedures if sterile technique was not correctly employed. Arterial lines were placed in a similar fashion using full barrier precautions.

8. All CVC and PICC insertion trays were augmented with components to comply with the central line bundle including the use of chlorhexidine sponges for cleaning the skin.

9. The infection preventionist gave feedback to the unit on adherence to protocol based on the central line dressing maintenance audits performed each month and any CLA-BSI every week. These audits assessed compliance with keeping the dressings clean, dry and intact. In addition, staff nurses were encouraged to practice proper line access techniques on a monthly basis by the nurse manager via emails and during staff meetings.

### Timely feedback on CLA-BSI occurrence and sentinel event investigation on CLA-BSI at the unit level - January 2010

Clinical Epidemiology provided weekly feedback on CLA-BSI occurrence to the unit leadership that included the ICU medical directors, CNS and nurse manager. The CNS responsible for each investigation was able to evaluate practitioner variation, nursing variation, CVC access and blood culture collection techniques, and anatomical CVC site of placement among other factors that may have contributed to each CLA-BSI. The nurse manager and medical directors shared the results of the investigation with the unit's nursing and medical staff, respectively.

### Positive reinforcement strategy - June 2010

We engaged hospital leadership to establish milestones for CLA-BSI avoidance (100, 200, and 365 days) and provide incentives upon achieving the milestones to sustain CLA-BSI improvement. These included coat pins indicating the days without a CLA-BSI and recognition breakfasts for the unit staff.

### Clinical epidemiologic investigation - November 2010

We conducted an epidemiologic investigation at the end of IY1 when, despite a modest improvement in CLA-BSI rates, there was a continued CLA-BSI burden in the face of implementation of and compliance with aggressive measures toward the CVC bundle and line maintenance practices. We investigated the types of organisms causing CLA-BSIs and the location of CLA-BSI patients within the ICU to evaluate for clustering of infections. Upon noting a surge and clustering of vancomycin-resistant *Enterococcus *(VRE) *faecium *CLA-BSIs from certain ICU rooms, Epidemiology performed environmental cultures of 42 high-touch surfaces (HTS) within the rooms where patients with VRE *faecium *CLA-BSI were identified. HTS cultured included bed rails, bedside tabletops, and keyboards, call buttons, supply cart handles and television remote controls.

### Molecular epidemiologic investigation **- **December 2010

Based on the results of the clinical epidemiologic investigation, a molecular epidemiologic investigation was conducted. Molecular typing of VRE *faecium *isolated from blood cultures and patients' environment was performed using repetitive extragenic palindromic sequence-based polymerase chain reaction (rep-PCR) DiversiLab kits (bioMérieux, Durham, NC, USA) following the Diversilab™ Enterococcus kit package insert and previously described methods [24-26]. An ATCC™ *E. faecalis *51299 strain was used as control along with another *E. faecium *control strain obtained from a patient's blood culture from a different area in the hospital. The modified Kullback-Leibler distance method was used to create a pairwise percent similarity matrix, and a dendrogram was generated using the unweighted pair group method of arithmetic averages. Isolates sharing greater than 97% similarity and/or indistinguishable (no band difference) were grouped for further analysis. The graph overlay feature was utilized to observe small differences between isolates that were otherwise not apparent on virtual gel images. Each new rep-PCR pattern identified was based on one peak difference and was assigned a sequential numeric classification based on the overlay. VRE organisms were considered similar if they had a one-peak difference.

### Environmental decontamination and nursing staff education - end of December 2010

Hospital policy already specified that all patients with VRE required contact isolation including a gown and gloves for anyone entering the room. Based on the results of the above investigation, in addition to continuation of contact isolation protocols an intensive terminal cleaning of all ICU rooms was conducted. Each pod of the ICU was emptied for a day at a time by moving patients into a different pod to facilitate this cleaning. The walls, floors and all surfaces and equipment in the rooms were spot cleaned and wiped down with a disinfectant. Environmental Services (EVS) personnel created a dedicated cleaning team that was specifically trained to clean the ICU rooms. Nursing staff was educated about proper technique for obtaining blood cultures by the clinical nurse specialists to reinforce their knowledge.

### Follow-up environmental culturing - March 2011

We repeated environmental culturing to document decontamination of VRE following deep cleaning of ICU rooms. Over 200 HTS from all ICU rooms were cultured for VRE to assess for continued effectiveness of cleaning by our EVS staff that were unaware of this surveillance.

### Chlorhexidine gluconate bathing - April 2011

We introduced CHG bathing of all patients in the ICU in response to one VRE CLA-BSI that occurred three months after environmental decontamination. This intervention was introduced to reduce the potential bacterial burden on patients [4]. Patients admitted to the ICU underwent a CHG bath on admission and daily with a diluted solution of 4% chlorhexidine gluconate in tap water based on previous studies showing eradication of VRE and methicillin-resistant *Staphylococcus aureus *(MRSA) colonization at this dose [27,28].

### Data collection

We measured CLA-BSI incidence per NHSN definition [29] before (baseline period) and after (post-intervention period) the implementation of the 'line bundle' and subsequent process improvement methods. The quarterly rate of infections was calculated as follows: (number of CLA-BSIs/number of central line days) × 1,000 for each three-month period. Quarterly rates were assigned to one of four categories based on when the study intervention was implemented: at baseline, during the early post-intervention period (year 1), or late post-intervention period (year 2). We also collected data on the number of temporary CVCs, including PICCs used in ICU patients over the study years. Device utilization ratio was calculated as follows in accordance with NHSN guidelines: number of device or catheter days/number of patient days [29]. Patient days were counted using the daily ICU census at midnight.

### Outcome measures and study hypotheses

Primary outcome measure was quarterly CLA-BSI rate per 1,000 central line days. Secondary outcome measures were compliance with CVC insertion and dressing maintenance practices. The primary study hypothesis was that the CLA-BSI rate would be reduced by at least 50% after implementation of the study intervention as compared to the baseline over a two-year intervention period. We did not evaluate the relative effectiveness of the separate components of the intervention.

### Statistical analysis

As used in previous studies [5], and because CLA-BSIs are rare events, a Poisson regression analysis was used to generate an incidence rate ratio (IRR) compared with baseline CLA-BSI rates (Stata software, version 10; Statacorp, College Station, TX, USA).

## Results

### CLA-BSI reduction

**Figure 1:**
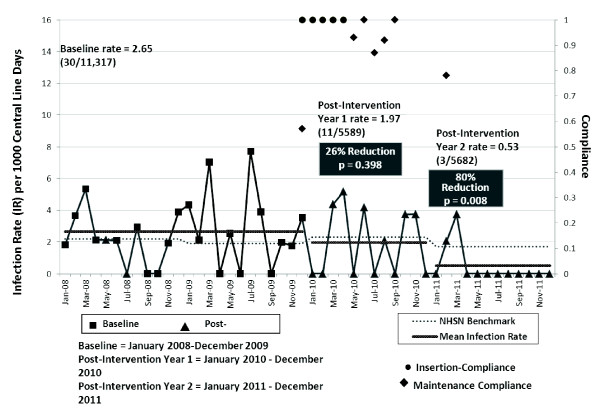
**Central line-associated bloodstream infections, compliance with central line insertion and dressing maintenance during the study period**. NHSN, National Health Safety Network.

There were 2.65 infections per 1,000 catheter days (30 CLA-BSIs in 11,317 central line days) in the ICU in the baseline period (Table 1). The net infection rate at the end of the two-year intervention period decreased by 53% to 1.24 infections per 1,000 catheter days (14 CLA-BSIs in 11,271 central line days) with IRR of 0.47 (95% CI = 0.25 to 0.88, *P *= 0.019) (Table 1). During IY1, the CLA-BSI rate was reduced to 1.97 per 1,000 catheter days (11 CLA-BSIs in 5,589 central line days); the IRR was 0.74 (95% CI = 0.37 to 1.65, *P *= 0.398). During IY2 (months 13 to 24 of the intervention), the CLA-BSI rate further reduced to 0.53 per 1,000 catheter days (3 CLA-BSI in 5,682 central line days); the IRR was 0.20 (95% CI = 0.06 to 0.65, *P *= 0.008) with 80% reduction compared to the baseline period. There were zero CLA-BSIs for the last 10 months of the intervention period included in the analysis (Figure 1) and with zero CLA-BSIs for a total of 15 calendar months.

**Table 1:** CLA-BSI incidence rate/1000 patient days, incidence rate ratio (IRR) in the post-intervention period compared to baseline period.

Surveillance period	Number of CLA-BSI	Central line days	CLA-BSI rate/1000 central line days	IRR (95% CI)	Percentage reduction*	*P *value
**Baseline (two-year period)**	30	11,317	2.65	NA	NA	NA
**Post-intervention year 1**	11	5,589	1.97	0.74 (0.37-1.65)	26%	0.398
**Post-intervention year 2**	3	5,682	0.53	0.20 (0.06-0.65)	80% (35%-94%)	0.008
**Post-intervention total**	14	11,271	1.24	0.47 (0.25-0.88)	53% (12%-75%)	0.019

*Compared to baseline period. CI, confidence interval; CLA-BSI, central line-associated bloodstream infections; NA, not applicable.

### Compliance with insertion, dressing maintenance, and line removal

Compliance with CVC insertion practices was high based on the audits; compliance with CVC dressing maintenance increased steadily and remained high at 80 to 100% during the intervention period assessments (Figure 1). Despite a daily checklist to remind the team of prompt removal of unnecessary CVCs, device utilization ratio (central line days/patient days) did not significantly change during the intervention (Table 2).

**Table 2:** Monthly CLA-BSI during the study period, device utilization ratio and organisms causing CLA-BSI in each month.

Surveillance period	Number of CLA-BSI	Central line days	CLA-BSI rate/1000 central line days	Patient days	Device utilization ratio	Organisms causing CLA-BSI
Jan-08	1	550	1.82	700	0.79	VRE *faecium*

Feb-08	2	547	3.66	675	0.81	*Acinetobacter baumannii* Methicillin-resistant *Staphylococcus epidermidis*

Mar-08	3	562	5.34	739	0.76	*Enterococcus faecium* Methicillin-resistant *Staphylococcus epidermidis* *Candida parapsilosis*

Apr-08	1	470	2.13	661	0.71	Group B *Streptococcus agalactiae*

May-08	1	468	2.14	668	0.70	Methicillin-resistant *Staphylococcus epidermidis*

Jun-08	1	478	2.09	629	0.76	*Enterobacter cloacae*

Jul-08	0	351	0.00	626	0.56	

Aug-08	1	340	2.94	683	0.50	*Klebsiella oxytoca*

Sep-08	0	360	0.00	649	0.55	

Oct-08	0	510	0.00	691	0.74	

Nov-08	1	518	1.93	696	0.74	*Pseudomonas aeruginosa*

Dec-08	2	514	3.89	699	0.74	*Klebsiella pneumoniae* Methicillin-resistant *Staphylococcus epidermidis*

Jan-09	2	461	4.34	700	0.66	*Prevotella buccae and Achromobacter xylosoxidans*

Feb-09	1	474	2.11	634	0.75	*Candida glabrata*

Mar-09	4	568	7.04	721	0.79	*Pseudomonas fluorescans, Peptostreptococcus*, VRE *faecium*

Apr-09	0	417	0.00	639	0.65	

May-09	1	394	2.54	644	0.61	*Candida albicans*, VRE *faecium*

Jun-09	0	358	0.00	626	0.57	

Jul-09	3	389	7.71	673	0.58	*Candida glabrata*, VRE *faecium, E.coli*

Aug-09	2	513	3.90	684	0.75	*Acinetobacter baumannii*, VRE *faecium*

Sep-09	0	436	0.00	661	0.66	

Oct-09	1	505	1.98	699	0.72	*Pseudomonas aeruginosa*

Nov-09	1	569	1.76	679	0.84	*Klebsiella pneumoniae*

Dec-09	2	565	3.54	728	0.78	*Enterococcus faecalis, Acinetobacter baumannii*

Jan-10	0	468	0.00	678	0.69	

Feb-10	0	411	0.00	626	0.66	

Mar-10	2	457	4.38	623	0.73	VRE *faecium*, VRE *faecium*

Apr-10	2	386	5.18	565	0.68	Methicillin-resistant *Staphylococcus epidermidis*, VRE *faecium*

May-10	0	480	0.00	615	0.78	

Jun-10	2	479	4.18	612	0.78	*Morganella morganii, Enterobacter cloacae*

Jul-10	0	468	0.00	654	0.72	

Aug-10	1	478	2.09	627	0.76	VRE *faecium*

Sep-10	0	423	0.00	626	0.68	

Oct-10	2	534	3.75	711	0.75	VRE *faecium*, VRE *faecium*

Nov-10	2	532	3.76	679	0.78	VRE *faecium*, Methicillin- resistant *Staphylococcus epidermidis*

Dec-10	0	473	0.00	620	0.76	

Jan-11	0	485	0.00	712	0.68	

Feb-11	1	485	2.06	647	0.75	VRE *faecium*

Mar-11	2	535	3.74	709	0.75	VRE *faecium, Enterococcus faecalis*

Apr-11	0	501	0.00	641	0.78	

May-11	0	461	0.00	677	0.68	

Jun-11	0	448	0.00	621	0.72	

Jul-11	0	351	0.00	655	0.54	

Aug-11	0	454	0.00	690	0.66	

Sep-11	0	543	0.00	705	0.77	

Oct-11	0	473	0.00	677	0.70	

Nov-11	0	473	0.00	653	0.72	

Dec-11	0	473	0.00	704	0.67	

CLA-BSI, central line-associated bloodstream infections.

### Clinical epidemiologic investigation

**Figure 2:**
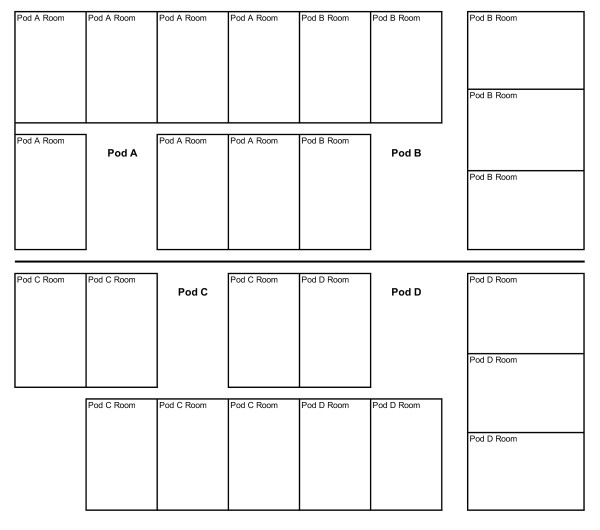
**Intensive care unit layout; four patients with VRE central line-associated bloodstream infection were located in two rooms of pod B - two in one room and two in another room**. One patient each was located in each of the other pods.

Seven out of eleven CLA-BSIs in 2010 were caused by VRE *faecium *(Table 2). Four out of seven patients with a VRE CLA-BSI were located in pod B; of the other three patients, one was in pod A, one in pod C and one in pod D (Figure 2). Out of the four patients with a VRE CLA-BSI in pod B, two were located in one room during different months; the other two were located in another room during different months. Selective environmental culturing for VRE of HTS was performed initially only in the rooms that harbored patients with a VRE CLA-BSI in 2010. Out of 40 HTS sites cultured in five patient rooms, 8/40 (20%) sites cultured positive for VRE *faecium*. The HTS that tested positive were bed rails (three cultures), supply cart handles (two cultures), computer keyboard (one culture), call button (one culture), and bedside table (one culture). Following deep environmental cleaning, all cultures were negative for VRE from all previously positive VRE patient rooms. Additional follow-up cultures of HTS in all ICU rooms performed four months later did not reveal any positive VRE.

### Molecular epidemiologic investigation

**Figure 3:**
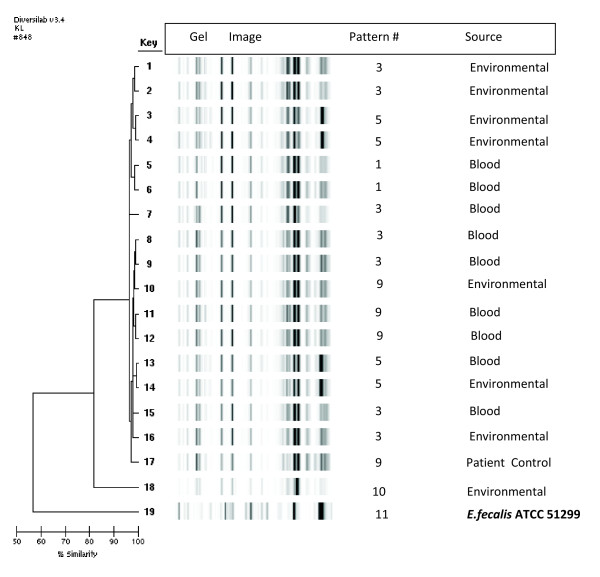
**Rep-PCR analysis of VRE isolates from blood cultures and environmental cultures related to patients with VRE CLA-BSI**. CLA-BSI, central line-associated blood stream infections; P, pattern number; VRE, vancomycin-resistant *Enterococcus faecium*. Control (row 19) = *E.faecalis *ATCC™ 51299 control strain.

The rep-PCR patterns of all VRE *faecium *isolates were depicted to be in Patterns 1 (Key numbers 5, 6), Pattern 3 (Key numbers 1, 2, 7, 8, 9, 15, 16), Pattern 5 (Key numbers 3, 4, 13, 14), Pattern 9 (Key numbers 10, 11, 12, 17), Pattern 10 (Key number 18) and Pattern 11 (Key number 19). Based on the overlay, Pattern numbers 1, 3, 5 and 9 had a single band difference and were related. Pattern numbers 10 and 11 were different from Pattern numbers 1, 3, 5 and 9. The *E. faecium *control strain obtained from a blood culture from a patient in a different area (Pattern 9; Key number 17) had a one-band difference from Patterns 1, 3, and 5. It was related to the patient and environmental samples under investigation. Control VRE *faecalis *strain ATCC™ 51299 (Pattern number 11; Key number 19) was unrelated to the patient and environmental samples under investigation (Figure 3).

### Sentinel event investigation of residual CLA-BSI

All seven residual CLA-BSIs were caused by VRE *faecium*. All VRE were cultured from one bottle from a CVC with one or more negative culture bottles from simultaneous peripheral blood cultures in all patients. Scenarios where blood cultures were obtained included: in response to hypotension caused by gastrointestinal bleed in two patients, pulseless electrical activity (PEA) in two patients (one after a radical neck dissection, one after transjugular intrahepatic portosystemic shunt (TIPS) procedure), one temperature recording of 100.8 in a patient with deep venous thrombosis (DVT), in response to leukocytosis in a patient who was afebrile and was treated with corticosteroids, and in response to leukocytosis in a patient with acute coronary syndrome. All seven patients had their catheters removed. Three patients died within three days of diagnosis of bacteremia secondary to their presenting diagnosis with none of the deaths attributed to the CLA-BSI. Four patients were treated for bacteremia for 14 days.

## Discussion

CLA-BSIs continue to be a challenge in healthcare delivery, especially in the ICU, where nearly 50% of patients will have a CVC inserted at some point in their care [30]. CLA-BSIs are responsible for significant morbidity resulting in extended hospitalizations, hospital costs accounting for a loss of up to $26,000 per CLA-BSI [31], and increased mortality accounting for approximately 100,000 deaths a year [1]. Though multiple investigators have reported interventions surrounding the central line bundle to successfully reduce the burden of CLA-BSIs in their units, few have reported the results of continued process improvements and secondary interventions that can be utilized when, as is often the case, compliance with the CVC bundle alone has not resulted in a significant reduction in these infections.

Our unit's culture in the baseline period likely mirrored what may be the standard culture in many institutions. Physicians and nurses were aware of the dangers of CLA-BSIs and educated on the expectation for strict adherence to sterile techniques for insertion and access of central lines. Physicians were expected to utilize full-barrier precautions and ultrasound guidance for CVC insertion. However, as with many academic institutions, there was a high turnover of new trainees into the environment making a sustained cultural change more difficult. Into this environment, our epidemiology team championed a systematic team-oriented approach to optimally reduce CLA-BSI to a near-zero rate. The implementation of a central line 'bundle' resulted in excellent compliance with sterile insertion techniques and substantial improvements in central line dressing care. However, despite improved compliance with the bundle, our initial efforts resulted in only a modest improvement of our CLA-BSI rate and the use of CHG Tegaderm™ dressings may or may not have contributed to this modest improvement; we were still far from our goal.

Our innovative team approach engaged hospital epidemiologists and clinicians with weekly CLA-BSI surveillance followed by immediate, unit-level, root cause analysis, which facilitated feedback to clinicians in a timely fashion when they still remembered the circumstances surrounding the infection. In the majority of our CLA-BSIs in IY1 we suspected potential blood culture contamination with VRE coupled with clinical deterioration from a noninfectious etiology as a likely cause of persistence of CLA-BSIs despite our compliance with bundle elements. Our interdisciplinary approach with infection prevention experts, critical care physicians and nursing staff working as one team facilitated an investigation that identified geographic clustering of VRE cases in our ICU. As VRE can cause both monoclonal and polyclonal outbreaks [32-35], we performed environmental cultures coupled with a molecular epidemiologic investigation based on our finding of geographic clustering and our suspicion of blood culture contamination. This documented environmental contamination with VRE and demonstrated the genetic similarity between environmental VRE and the VRE associated with these CLA-BSIs. We then implemented a very aggressive intervention of cleaning our ICU by systematically emptying one ICU pod at a time and engaging our Environmental Services team in our process improvement strategy. This investigation also recognized deficiencies in the structure of our environmental cleaning plan and identified the need to have more highly trained cleaning staff that was dedicated to an ICU. As a result, we have now employed environmental cleaning teams that are dedicated to ICUs and are trained to clean around ICU equipment in our institution. Studies have shown elimination of VRE colonization events by meticulous attention to environmental cleaning [36,37], but very few have utilized environmental decontamination as an intervention to achieve a reduction in VRE CLA-BSIs. We observed a decline in our VRE CLA-BSI upon implementation of meticulous environmental cleaning. We chose not to conduct admission and/or weekly surveillance cultures for VRE colonization followed by contact isolation as an intervention as it is an expensive strategy. Instead, based on our investigation and findings, we focused on improving environmental cleaning and reducing potential bacterial burden on our ICU patients [4,27,28].

Based on our experience, there can be an underlying cause for reminiscent CLA-BSIs after implementation of a process improvement project emphasizing bundle compliance. How to deal with these residual infections is not frequently reported in the literature. There are many potential interventions including patient cohorting, purpose-made catheter sterilizing devices, dedicated line teams or, as in our case, environmental decontamination to name a few. Deciding which intervention to use next could be difficult and using all or some interventions randomly can be costly. We suggest that unit leaders should investigate the root cause of their residual infections rather than implementing further measures piecemeal.

This work demonstrates the success of this positive approach to the issue of CLA-BSIs, investigating the underlying cause of reminiscent CLA-BSI thereby sustaining CLA-BSI reduction. We have demonstrated that with an iterative team approach and by eliminating underlying causes of residual CLA-BSI and basing our approach on internal evidence for the need for further interventions, CLA-BSI reduction is possible and sustainable. Our efforts to reduce CLA-BSI incidence demonstrate a synchronized model for multidisciplinary teams, which included a hospital epidemiologist and administrative leaders to increase compliance with bundle elements and to decrease blood culture contamination. We engaged hospital leadership to establish milestones and recognize the unit at the institutional level upon milestone achievement. This strategy resulted in a positive effect on morale, and facilitated staff compliance with the new standards of care. At the same time, it promoted the culture of safety: no one wanted to be the practitioner that started the clock back to zero days since the last CLA-BSI. We believe that these two strategies played an important role in sustaining our success with CLA-BSI reduction.

We observed a stability of the device utilization ratio, which may be due to the fact that a patient with multiple lines; for example, a triple-lumen CVC, and dialysis catheter counts as one line day, even with discontinuation of one of the lines the patient would still count as a central line day. Thus the device utilization ratio may underrepresent how many lines were being removed even with aggressive removal based on a daily goals checklist. While the burden and exposure may be reduced, the device utilization ratio will not account for this. This may argue for counting the number of lines or even lumens for a lumen/patient/day count. Unfortunately, this may be unfeasible in most settings leading to inaccurate counts. However, improved line day counts might be possible in future studies as electronic medical record tracking of vascular access days in ICUs increases.

Our experience also validates the existent concerns that all CLA-BSI may not be preventable given the high sensitivity and low specificity of CDC/NHSN definition, as some of these positive blood cultures with significant pathogens may be related to contamination or catheter colonization [20]. This may especially be true in units colonized with resistant bacteria such as VRE or MRSA. Eventually, these units may be penalized financially if, as planned, healthcare providers in the United States receive only limited reimbursement from the Centers for Medicare and Medicaid Services (CMS) for any CLA-BSI acquired in the hospital, since this is now accepted as 'preventable' [21]. An acceptable compromise may envision CMS accounting the reasons why the reminiscent CLA-BSIs were not preventable on a case-by-case basis (for example, an 'infection' per definition may not be an infection at all or the 'infection' may be a secondary bloodstream infection with another potential primary site) instead of basing reimbursement merely on CLA-BSI rate per institution.

Our study does have several limitations. First, we studied one particular ICU population with a homogeneous group of nurses and house staff. Thus, our results may not be extrapolated to surgical, cardiac, or other ICU populations. However, the principle of our process is the continuous reporting of CLA-BSIs and refinement of unit-specific interventions, which should be reproducible in other ICU populations. Second, we did not continuously monitor central line insertion and dressing maintenance throughout the intervention period. Even at baseline, the compliance with sterile technique for insertion was extremely high and in addition, these audits were labor intensive and we felt that intermittent auditing was an acceptable alternative to continuous audits once compliance with the sterile line insertion techniques and dressing maintenance had been achieved. In addition to intermittent audits, we continue to conduct educational sessions with periodic (yearly) reinforcement of education related to central line maintenance and access. Our CNSs continued to investigate any new CLA-BSIs including assessment of line access practices and insertion technique. In addition, house staff trainees are given a half-day training session in our simulation laboratory emphasizing central line placement prior to each rotation in the ICU. Third, we only cultured the ICU rooms where patients were diagnosed with VRE CLA-BSI during the first round of environmental culturing. However, we felt the high prevalence of VRE on HTS in the rooms cultured justified the deep cleaning of every room in the unit. In addition, we cultured all HTS in all ICU rooms during our follow-up environmental culturing to document decontamination of the rooms post environmental cleaning and to assess continued effective cleaning by our environmental staff. Fourth, we monitored hand hygiene upon entry and exit of the rooms through audits performed by the unit staff, which showed very high compliance of greater than 90% during early months of the study, but we did not monitor whether staff cleaned hands prior to accessing central lines. Doing such audits anonymously for accurate data gathering would be a challenge but we believe that with increased awareness of CLA-BSI prevention, this practice improved over time during this study. Last, we did not evaluate the relative effectiveness of the separate components of the intervention related to central line bundle. However, our goal was to attain maximal improvement of patient safety in our ICU; this quality improvement initiative was designed to optimize the use of well-documented best practices and offered the greatest probability of reducing CLA-BSI incidence. We saw a stepwise decline in CLA-BSIs as we strategically introduced accessory interventions beyond the central line bundle. It remains unknown whether implementation of CHG bathing alone or environmental cleaning alone (given enough time) in the absence of the other would have eliminated reminiscent sporadic CLA-BSI in our ICU. However, we believe that deep environmental cleaning along with correction of deficiencies in the daily environmental cleaning for ICUs was necessary to reduce bacterial burden in the environment surrounding the patients in our ICU. We saw a decline in VRE CLA-BSIs after this intervention but extended the decontamination approach to patients with another occurrence of VRE CLA-BSI since our follow-up cultures did not reveal VRE on multiple HTS. Our approach demonstrates our zero tolerance for CLA-BSI occurrence in our ICU. We also validated the currently existing thoughts about the high sensitivity and poor specificity of CDC/NHSN definition for CLA-BSI. We showed that multidisciplinary efforts toward clinical epidemiologic investigation could actually lead to a near-zero rate even in a complex ICU in a tertiary care hospital such as ours despite these concerns.

It may be argued, in light of the fact that the majority of our residual CLA-BSIs were suspected to be contaminants, that our improvement comes at a significant resource cost and that we implemented aggressive interventions to try to curtail nonexistent infections in the setting of contamination; however, we submit that these were reasonable interventions since we suspected central line contamination which, in the setting of high environmental burden, reflects the risk of impending invasive infections unless action is taken. In addition, it is difficult for physicians to choose not to treat a patient who has a positive blood culture with a significant pathogen. Retrospectively, we suspected that the majority of these cultures were contaminants; however, the treating team presented with the culture results on critically ill patients in real time had to treat the culture as a real result. Thus the elimination of these potentially erroneous CLA-BSIs has the added effect of reducing unnecessary antibiotic use. Unnecessary antibiotic use, however, can lead to increased hospital length of stay among other complications thus making it important to eliminate blood culture contamination via both compliance with line insertion bundle and environmental decontamination. We encouraged clinicians to avoid drawing blood cultures from the existing catheters at the same time as we instituted these interventions. However, we saw improvement in CLA-BSI before there was a change in this behavior. We are slowly seeing a change in this behavior and are continuing to work on changing the culture of drawing blood cultures from existing CVCs. We felt that it was important to address the recognized deficiencies in the structure of our environmental cleaning plan and address the environmental contamination as soon as we identified the problem.

## Conclusions

In conclusion, strict adherence to the central line bundle is essential to prevention of CLA-BSIs, but may not completely eliminate these infections as blood culture contamination contributes to CLA-BSIs that are detected by CDC/NHSN surveillance definition. Efforts to further reduce residual CLA-BSIs require a strategic multidisciplinary team approach focused on epidemiologic investigations of practitioner or unit-specific etiologies. Continuous process improvement can then be targeted at local factors contributing to a CLA-BSI, such as environmental contamination in our case, with evidence-based interventions. Sustained reduction of CLA-BSIs requires longitudinal support of hospital and unit leadership to continue to improve the care of our most vulnerable ICU patients.

## Key messages

Our study methods and findings include:

• Use of a multidisciplinary team including Clinical Epidemiology for root cause investigations.

• With reeducation and reemphasis on the central line bundle we achieved modest reductions in our rate of central line-associated bloodstream infections (CLA-BSIs) during our first year.

• During the second year, we utilized a root cause analysis approach to investigating our CLA-BSIs leading to the discovery of environmental contamination and eradication of CLA-BSIs with patient chlorhexidine bathing and deep environmental cleaning.

• Use of PCR techniques to confirm environmental contamination as the source of CLA-BSIs.

• Discussion of the CDC definition of CLA-BSI that includes inclusion of potentially contaminated line cultures in the calculated rate of CLA-BSIs for the unit.

## Abbreviations

CDC: Center for Disease Control; CHG: chlorhexidine gluconate; CLA-BSI: central line-associated bloodstream infections; CMS: Centers for Medicare and Medicaid Services; CNS: clinical nurse specialists; CVC: central venous catheters; DVT: deep venous thrombosis; EVS: Environmental Services; HAI: healthcare-associated infections; HTS: high-touch surfaces; ICU: intensive care unit; IRR: incidence rate ratio; IY: intervention year; MRSA: methicillin-resistant *Staphylococcus aureus*; MSB: maximal sterile barriers; NHSN: National Health Safety Network; IHI: Institute for Healthcare Improvement; PA: pulmonary artery catheters; PEA: pulseless electrical activity; PICC: peripherally inserted central catheters; rep-PCR: repetitive extragenic palindromic sequence-based polymerase chain reaction; TIPS: transjugular intrahepatic portosystemic shunt; VRE: vancomycin-resistant *Enterococcus*.

## Competing interests

The study was partially funded by a quality improvement grant from Cardinal Health Foundation. HL095772 to MCE. JEM received $20,000 research grant to study UV disinfection from Medline Industries Inc. and received $3000 in honorarium for lectures on Making a Difference in Infectious Diseases Pharmacotherapy.

## Authors' contributions

MCE participated in conceptual design, in executing the project, data analysis and drafted the manuscript. NAA participated in conceptual design, in executing the project, and helped draft the manuscript. NZ participated in data gathering, in executing the project, and helped draft the manuscript. JEM participated in conceptual design, in executing the project, and helped draft the manuscript. KT participated in conceptual design, executing the project, and helped draft the manuscript. BV participated in conceptual design, executing the project, and helped draft the manuscript. JSC participated in conceptual design, executing the project, and helped draft the manuscript. ML participated in study design and performed statistical analysis. PP participated in conceptual design, performed molecular epidemiologic investigation, and helped draft the manuscript. MMS participated in conceptual design, in executing the project, clinical and molecular epidemiologic investigation, data analysis and drafted the manuscript. All authors read and approved the final manuscript.
